# Feasibility and acceptability of an implementation strategy to enhance use of classroom-based physical activity approaches in elementary schools: a mixed methods study

**DOI:** 10.1186/s12889-025-25333-0

**Published:** 2025-11-27

**Authors:** Timothy J. Walker, Derek W. Craig, Christopher D. Pfledderer, Matthew Lee, Kempson Onadeko, Emma E. Saving, John B. Bartholomew, Maria E. Fernández

**Affiliations:** 1 Center for Health Promotion and Prevention Research, The University of Texas Health Science Center at Houston School of Public Health, Houston, TX USA; 2 Department of Health Promotion and Behavioral Sciences, University of Texas Health Science Center at Houston School of Public Health Austin Location, Austin, TX USA; 3 UTHealth Houston Institute for Implementation Science, The University of Texas Health Science Center at Houston School of Public Health, Houston, TX USA; 4 Department of Kinesiology, Penn State University, University Park, PA, USA

**Keywords:** Classroom-based physical activity, Implementation strategy, Feasibility, Acceptability, Teacher training, Leadership training, Mixed methods

## Abstract

**Background:**

Classroom-based approaches are an evidence-based way to improve children’s physical activity; however, they are inconsistently implemented in schools. We developed MAGIC (Movement for Academic Growth In Classrooms), a multifaceted implementation strategy that consists of leadership training, teacher training, and a monthly newsletter to improve use of classroom-based physical activity approaches. The purpose of this study is to examine the feasibility and acceptability of MAGIC among elementary school teachers and leaders.

**Methods:**

We used an embedded, convergent mixed methods design. We provided the MAGIC implementation strategy to a partner elementary school. We then administered surveys to teachers to quantitatively examine feasibility and acceptability. The surveys included questions about training attendance, receipt of newsletters, and acceptability based on a theoretical framework of acceptability. We used descriptive statistics to understand feasibility and acceptability trends. We also conducted semi-structured group and individual interviews with teachers and school leaders to understand perspectives about MAGIC components. We used rapid assessment procedures to analyze qualitative data, and multiple integration strategies, including joint displays, to compare quantitative and qualitative findings.

**Results:**

A total of 35 teachers (kindergarten-5^th^ grade) completed study surveys and 27 teachers and school leaders participated in interviews. As an indicator of feasibility, all leaders attended leadership training sessions 1 (*n* = 13/13), 2 (*n* = 13/13), and 4 (*n* = 7/7); and 57% attended session 3 (*n* = 7/13); 93% (*n* = 28/30) of teachers attended training session 1, 90% (*n* = 27/20) attended session 2, and 87.5% (*n* = 21/24) attended session 3; and 67–75% of teachers received respective newsletters. The trainings and newsletters had high acceptability levels as indicated by survey results and interview data. Trainings included flexible scheduling integrated into existing meetings, which participants reported helped improve feasibility. Participants also highlighted the importance of incorporating classroom-based approaches into trainings and the simplicity of the newsletter, which contributed to their acceptability.

**Conclusions:**

This study provides evidence supporting the feasibility and acceptability of the MAGIC implementation strategy among elementary school teachers and leaders. Future research should test MAGIC among more elementary schools to examine its impact on teacher implementation outcomes and students’ physical activity levels.

**Trial registration:**

ClinicalTrials.gov, NCT05048433, registered 9/8/2021, https://clinicaltrials.gov/ct2/show/NCT05048433

**Supplementary Information:**

The online version contains supplementary material available at 10.1186/s12889-025-25333-0.

## Background

Schools are an important setting for physical activity promotion among children [1]. Despite this, many school-based physical activity interventions have limited effectiveness and often fail to produce sustainable impacts on children’s moderate-to-vigorous physical activity [2–5], which contributes to a majority of children not meeting physical activity guideline recommendations[6]. The lack of success can be due to ineffective components, poor implementation, compensatory behaviors, and the pressure on schools to prioritize standardized testing outcomes [3–5, 7]. Additionally, school-based physical activity interventions are often complex and not feasible to sustain without strong leadership support given competing priorities and limited resources [8].

Classroom-based approaches are a promising way to support students’ physical activity because children spend most of their school day in classroom instruction [9]. They can be used in different ways (e.g., during transitions, to energize students, or as part of structured lessons) to both enhance classroom management and support learning, which further aligns them with schools’ goals of improving academics [10]. There are two common forms: physically active breaks (short bouts of activity used in between classroom lessons) and physically active lessons (integration of movement into learning activities as part of academic lessons) [11, 12]. Systematic reviews and randomized controlled trials support their effectiveness for improving children’s physical activity [13–16], academic achievement [17], and on-task behavior [18]. Educators also report classroom-based approaches support children’s learning readiness, classroom engagement, and behavior, which collectively can contribute to academic achievement [19].

Despite their promise, classroom-based physical activity approaches are inconsistently implemented in practice, especially in elementary schools [20, 21]. Studies have identified multiple implementation barriers including a lack of time, low levels of knowledge, and low self-efficacy among teachers; and competing priorities, a lack of leadership support, unsupportive school cultures, and limited professional development opportunities at the school level [21–25]. Given the numerous implementation challenges and inconsistent implementation, there is a need to develop and test implementation strategies to improve the uptake, use, and sustainment of classroom-based physical activity approaches among elementary schools.

To address this need, our team used Implementation Mapping to co-develop an implementation strategy for classroom-based physical activity approaches in elementary schools [26]. Implementation Mapping is a systematic process that uses partner input, theory, and empirical evidence to guide implementation strategy design and testing. Using this process, we developed MAGIC (Movement for Academic Growth In Classrooms), which aims to shift perspectives about the role of physical activity in schools by helping educators use physical activity in purposeful ways to enhance learning, behavior, and health [27]. MAGIC is a multifaceted implementation strategy that includes leadership trainings, teacher trainings, and a monthly newsletter. Collectively, the MAGIC components are designed to enhance leadership support and build a sustainable culture around the strategic use of physical activity, while also building teacher skills and self-efficacy. Trainings are designed to be co-led by district and school leaders to build capacity while also providing direct implementation support [27]. This capacity building approach is designed to support real-world implementation (i.e., the use of existing evidence-based practices) and address challenges during scale up [28].

Traditionally, implementation strategies such as teacher trainings have been used to enable implementation as part of testing the effectiveness of interventions for classroom-based physical activity approaches [29, 30]. More recently, studies have included implementation strategies with program materials [31], which is consistent with a designing for dissemination approach [32]. Studies have also taken a greater focus on testing the acceptability, feasibility, and effectiveness of teacher trainings for implementing classroom-based physical activity approaches, which have had mixed success (i.e., some trainings have been effective and acceptable, whereas others lack evidence of preliminary effectiveness and acceptability) [33–36]. However, there are limited studies that focus on developing multifaceted implementation strategies to support classroom-based physical activity approaches. MAGIC was designed to address this gap by targeting the implementation barriers that teachers, leaders, and schools commonly face.

Given MAGIC represents a new approach to supporting classroom-based physical activity, it is critical to examine its feasibility and acceptability. A key purpose of feasibility testing is to determine whether an “intervention” (or in this case, the MAGIC implementation strategy) should be recommended for further testing [37]. An essential aspect of feasibility testing, is to examine acceptability, or how the intended individuals react to the intervention (i.e., the MAGIC implementation strategy). Therefore, the purpose of this study is to examine the feasibility and acceptability of the MAGIC implementation strategy among teachers and school leaders.

## Methods

### Parent study

This study was part of a larger parent study that developed the MAGIC implementation strategy using Implementation Mapping, and examined MAGIC’s preliminary impact on teacher implementation fidelity of classroom-based approaches [38]. The parent study worked with two elementary schools: one receiving the MAGIC implementation strategy and the other serving as a control. As part of the parent study, teachers completed surveys at baseline (fall of 2023), follow-up 1 (spring 2024), and follow-up 2 (fall 2024). Teachers and school leaders (principal, assistant principal, and instructional coaches) also participated in interviews in the spring of 2024 and spring of 2025.

### Study design

**Fig. 1:**
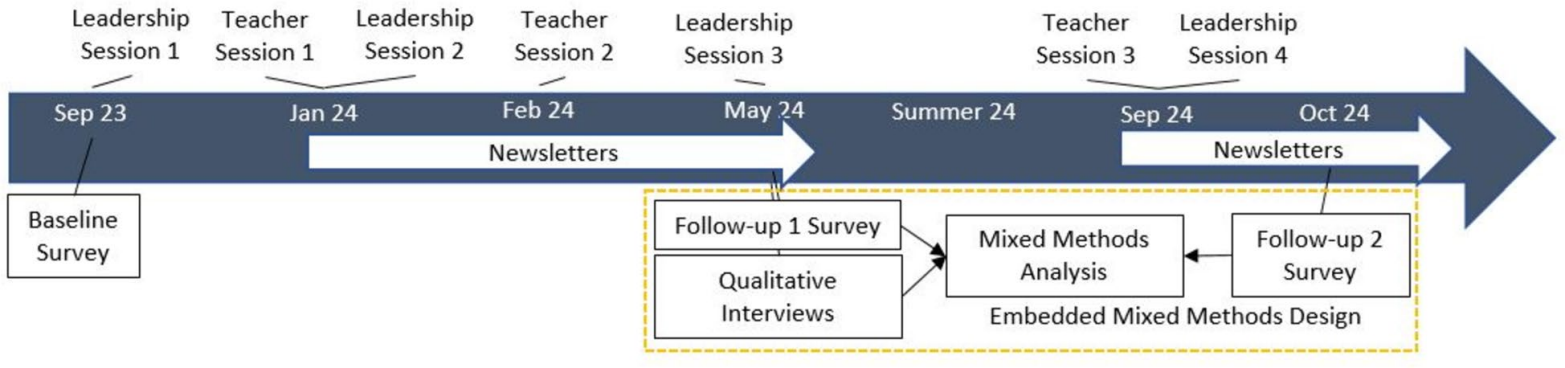
Study timeline and embedded convergent mixed methods design

This study used an embedded, convergent mixed methods design to attain a comprehensive understanding of the acceptability and feasibility of the MAGIC implementation strategy among teachers and staff. Specifically, we used quantitative survey data from the follow-up surveys, and qualitative interview data from teachers and school staff (Fig. 1). The survey and interview data were collected as part of the parent study, and included additional questions related to the acceptability and feasibility of MAGIC, which we used in the current study. More specifically, we used the quantitative data to describe trends related to feasibility and acceptability, and the qualitative data to expand on the quantitative data to provide a comprehensive understanding of teachers and school leaders’ opinions of the MAGIC implementation strategy. The Committee for Protection of Human Subjects at UTHealth Houston School of Public Health and the partner district’s department of research and accountability approved study procedures.

### Implementation strategy

We co-produced the MAGIC implementation strategy using Implementation Mapping. MAGIC was informed by Social Cognitive Theory and the R = MC^2^ heuristic (Readiness = Motivation x Innovation Specific Capacity x General Capacity) [39, 40]. Thus, MAGIC is theoretically informed, and designed to address common implementation barriers such as leadership support and teacher’s self-efficacy. MAGIC helps school leaders launch the use of classroom-based approaches, establish a supportive culture, and sustain teacher’s use. MAGIC is also designed to motivate teachers/staff, build skills and self-efficacy, and foster support among teachers. MAGIC includes three key components: 1) leadership trainings, 2) teacher/staff trainings, and 3) a monthly newsletter. The leadership trainings are delivered first, followed by teacher trainings and newsletters (Fig. 1). A distinctive feature of all training sessions was the use of physical activity to support learning. The newsletter was distributed to teachers/staff after the trainings to reinforce content and provide links to existing, online implementation resources. Details about the development of MAGIC and a complete Implementation Mapping (IMap) Logic Model showcasing its proposed mechanisms of action are published elsewhere [27].

MAGIC includes four leadership training sessions (with a focused leadership team) and three teacher training sessions (Supplemental Table 1), based on partner preference. We used a co-facilitation approach for trainings that included a study team member with a background in health promotion and behavioral sciences, a former district-level wellness coordinator with an education background, and the partner district’s Health & Physical Education Coordinator. A classroom teacher from the partner school also helped co-facilitate the final teacher session. We delivered leader and teacher training sessions in person, using PowerPoint slides. All sessions included the same general format, which started with a group movement activity (similar to a physically active break), followed by the key session content (delivered both without activity and with activity, similar to a physically active lesson), and closing with a review activity. This format allowed us to model both physically active breaks and lessons throughout the training sessions. The monthly newsletters were also co-produced by the study team and the district’s Health & Physical Education Coordinator using Canva. After co-creating newsletters, we shared them with the school principal to distribute to school staff (Supplemental Table 1). Collectively, MAGIC was designed to help each phase of implementation: 1) adoption, deciding to initiate classroom-based physical activity approaches; 2) implementation, using classroom-based approaches with students; and 3) maintenance, continuously using classroom-based approaches over time.

### Participant recruitment

As part of the parent study, we recruited two elementary schools in southeast Texas in the summer of 2023 by presenting the study at a district-wide leadership training. After identifying school partners, we presented the study at partner schools during regularly scheduled staff meetings to recruit classroom teachers (kindergarten-5th grade). Our goal was to recruit all classroom teachers at our partner schools to participate in the study. We also met individually with teachers to answer questions about the study and obtain written consent. By consenting, teachers agreed to complete surveys and implementation logs throughout the study. In the spring of 2024, we also invited teachers and school leaders (i.e., principal, assistant principal, and instructional coaches) to participate in group or individual interviews. We obtained written consent prior to completing each interview. The current study focused on the sample of teachers and school leaders at the school that received the MAGIC implementation strategy.

### Data collection

#### Quantitative data collection

We developed the teacher survey in Qualtrics and distributed it via email using individualized links. We reminded teachers to complete surveys through email and during on-site data collection by research staff as part of the parent study (i.e., student physical activity data). Teachers received a $20 gift card for completing each survey. This study used data from baseline (fall 2023), follow-up 1 (spring 2024), and follow-up 2 (fall 2024) surveys from the teachers at the intervention school (Fig. 1). We used demographic data from baseline surveys (e.g., gender, age, years of experience, grade-level, educational training, race, and ethnicity) and data from questions related to training attendance, receipt of newsletters, and acceptability of the implementation strategy from follow-up surveys. Training attendance and receipt of newsletters provided an indication of the feasibility to reach teachers through these strategies. If teachers reported attending a training and/or receiving a newsletter, they answered corresponding acceptability questions. These questions were informed by a multi-construct theoretical framework of acceptability [41], and were adapted from previous research [35]. The questions asked about affective attitude, coherence, effectiveness, and burden of the implementation strategy components (Supplemental-Table 2). We also included questions about whether teachers would recommend the training and benefit from more support.

#### Qualitative data collection

Two research team members with backgrounds in public health and qualitative training (TW and DC) conducted in-person, semi-structured group and individual interviews in Spring 2024 (same time period as follow-up 1 surveys). We completed group interviews by attending regularly-scheduled teacher meetings for respective grade-levels. For teachers unable to attend group interviews, we arranged time to complete an individual or small group interview. We opted to start with group interviews based on partner input and because this allowed us to efficiently connect with most teachers. We also completed individual or small group interviews with school leaders (i.e., principal, assistant principal, and instructional coaches). The interview guide included questions about teacher’s opinions of the trainings and newsletters (Supplemental-Table 2). For school leaders, the interview guide focused on how they supported teachers and their opinions about leadership trainings, teacher trainings, and newsletters. On average, interviews lasted 39 min in duration. All participants received a $30 gift card for completing an interview. All interviews were audio recorded and transcribed using an automated transcription service (Rev). Study team members checked the quality of each transcript prior to analysis.

### Data analysis

#### Quantitative analysis

We examined descriptive statistics (means, standard deviations, and frequencies) of demographic variables from the survey samples. We also examined frequencies of training attendance (as reported on the survey) and acceptability items. Notably, school leaders did not complete surveys, so attendance data were collected during each session and the acceptability analyses for leadership trainings were based on qualitative data only. We used StataSE 15 for data processing and the quantitative analysis.

#### Qualitative analysis

We used rapid assessment procedures to examine qualitative data, which is a team-based approach [42]. We created interview summaries for each interview transcript using Microsoft word. The summaries were organized by the interview guide and included bulleted information from each interview within each question domain. We then created matrices in Microsoft excel by including the question domains (e.g., opinions about training, opinions about newsletter) across the columns and the interview type (e.g., interview with kindergarten teachers) down the rows. We reviewed the information within each domain by interview type, first inductively, to highlight trends across interviews. We then deductively examined the domains using the categories from theoretical framework of acceptability [41] (e.g., affective attitude, coherence, effectiveness). We synthesized data from each domain to provide an in-depth understanding. Additionally, the inductive coding highlighted a series of recommendations to improve the strategy, for which we analyzed thematically. We shared back preliminary results with our district partner and a classroom teacher who participated in an interview to help verify accuracy.

#### Mixed methods analysis

We used multiple mixed methods integration strategies for this study [43]. The qualitative data were from the same teachers who completed the survey. Qualitative interview questions addressed each implementation strategy component (trainings and newsletter), and were designed to expand on the quantitative survey questions to gain a better understanding of the acceptability and feasibility of MAGIC. During analyses, we merged the survey items with qualitative interview domains to facilitate comparison and provide an in-depth understanding of acceptability across domains for teacher trainings and the newsletter. Additionally, we presented quantitative and qualitative results using side-by-side joint displays, which further facilitated comparisons.

## Results

### Study sample

During the first study year, all eligible classroom teachers (kindergarten- 5th grade) at the intervention school (*n* = 30) provided consent to participate in the survey portion of the study. All 30 classroom teachers completed the survey at follow-up 1, and 23 classroom teachers and four school leaders participated in interviews. Participants were mostly female, non-Hispanic, and either Black or White, and all grade-levels were represented within both samples (Table 1). During the second study year, 11 teachers left the school or shifted positions (e.g., transitioned to pre-k, special ed) and there were six new classroom teachers. Five (of the six) new teachers consented to be in the study and completed the follow-up 2 survey. Thus, 24/24 eligible teachers completed the survey at follow-up 2. Descriptive statistics for the respective study samples are provided in Table 1.

**Table 1:** Descriptive statistics for survey and interview samples

**Variables**	**F/U 1 Survey** *n* = 30	**F/U 2 Survey** *n* = 24	**Interview** *n* = 27^*^
Age in years, mean (SD)	36.6 (11.5)	37.8 (12.2)	35.5 (10.4)
Years in Current Positions, mean (SD)	5.8 (7.6)	6.5 (6.3)	5.1 (6.8)
Years as Teacher, mean (SD)	8.5 (8.0)	10.2 (8.7)	8.2 (7.7)
Sex, *n* (%), Female	26 (87)	22 (92)	24 (89)
Grade, *n* (%)			
Kindergarten-2nd Grade	14 (46.7)	14 (58.3)	11 (40.7)
3rd-5th Grade	16 (53.3)	11 (45.8)	12 (44.4)
School leaders	0 (0)	0 (0)	4 (14.8)
Race, *n* (%)			
Black or African American	17 (56.7)	15 (62.5)	15 (65.2)
White	11 (36.7)	8 (33.3)	7 (30.4)
Another race	2 (6.7)	1 (4.2)	1 (4.3)
Ethnicity, *n* (%), non-Hispanic	23 (76.7)	18 (75.0)	20 (86.9)

^*^Four participants had missing data for years in current position, years teaching, race, and ethnicity

### Feasibility of leadership trainings, teacher trainings, and newsletter

There were four leadership sessions in total (Supplemental Table 1). The first, third, and fourth leadership sessions were held during regularly scheduled leadership meetings (during the school day). The second leadership training session occurred during an in-school professional development day. Leadership training sessions were about 45 min in duration and attendance ranged from 58–100% (Table 2). The final leadership session, which occurred in fall 2024, included fewer leaders (7 instead of 13) because the district reduced staff members for the 2024–25 school year due to budget cuts. Leaders chose to have sessions during their regularly scheduled meetings and interview participants highlighted the need to be flexible with scheduling given the possibility of unexpected issues arising during the school day. Notably, one leadership training session was rescheduled due to an unexpected event.

**Table 2:**
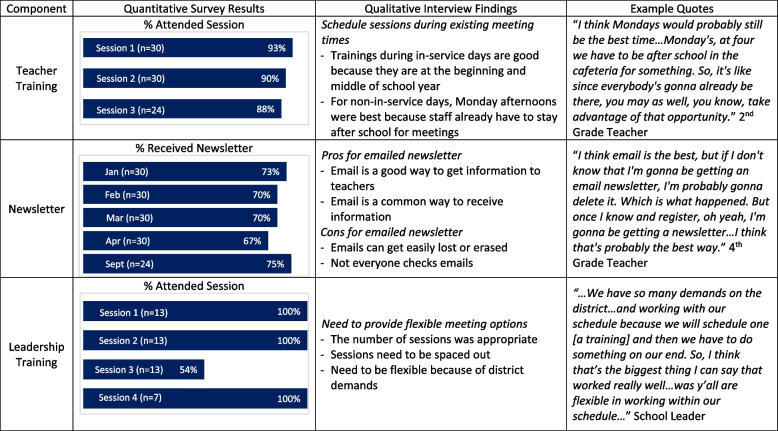
Feasibility joint display

There were three teacher trainings in total (Supplemental Table 2). The first was held during a professional development day in January 2024, before the start of the spring term. The other two teacher trainings were held during regularly scheduled staff meetings on Monday afternoons after school (one in February 2024 and the other in September 2024). The teacher trainings were approximately 45–60 min long and even though they were designed primarily for teachers, the entire school staff, including leaders, attended the sessions. Of the classroom teachers, ninety percent (*n* = 27/30) attended the two sessions in year one, and 87.5% (*n* = 21/24) attended the training session in year two (Table 2). Interview participants commented that they preferred having trainings during the teacher in-service days because they were tired after the long school day. Teachers also stated that if trainings were held during the week, then they should be held during the regularly scheduled staff meetings.

The percentage of teachers who reported receiving newsletters ranged from 66.7–75.0% (Table 2). Interview participants noted that email was a good way to send the newsletter. However, one teacher explained that it would be helpful to better alert teachers about the newsletters (e.g., sending a paper flyer), so they would know to check their email. Participants noted that not everyone checks emails and that some emails get lost or erased easily, which they suggested as a reason why some people may have not received newsletters.

### Leadership training acceptability

Given fewer leaders were involved with trainings compared to teachers, acceptability results focused on interview data only. Interview participants highlighted that leadership sessions were slow to start, but once things “got moving,” they enjoyed the sessions (Table 3). Leaders felt the sessions were progressive and they liked how physical activity was incorporated in leadership sessions. Leaders also reported the sessions helped them understand the need for movement and included helpful ways to talk with teaching teams about the need for movement. Leaders also commented on the importance of showing data in the sessions and that getting buy-in from leaders was important for getting teacher buy-in. Overall, leaders reported the number of sessions and length to be appropriate.

**Table 3:** Acceptability of leadership trainings

Domain	Qualitative Interview Findings	Example Quotes
Affective Attitude (How an individual feels about trainings)	- Started slow and gradually progressed - Got leaders moving and enjoyed it - Enjoyed the new activities demonstrated in sessions	“*Cause y'all brought really good things. The activities that you had us do…the boost activities you guys had us do. Like, I had never seen those before. So that was a breath of fresh air.*”
Effectiveness (Extent to which trainings are perceived as achieving their purpose)	- Important for leaders to understand the need for movement - Important to get buy-in from leaders - Giving information so leaders can talk to teams worked well - Showing data & providing background information worked well	“*Uh, the fact y'all used some of the strategies on us and had us do some of the activities in there too, because you have to get the leadership team to buy in before you get the campus to buy in.*” School Leader
Burden (Perceived effort required to participate in trainings)	- The number of leadership sessions were appropriate - Leadership team catches on quicker - If providing more sessions, they need to be shorter	“*I think that's the appropriate length…45 min to an hour. If you go over that, you're gonna start really losing people.*”

School leaders did not directly address the ease of understanding leadership training content within the interviews

### Teacher training acceptability

*Affective Attitude (how an individual feels about the training)*. Eighty-nine percent (in year 1) and 90.5% (in year 2) of teachers reported they enjoyed the trainings (Table 4). Interview participants explained they liked how physically active games were incorporated in trainings, which made the sessions fun and engaging. They also commented that trainings helped boost team morale and were a nice break from traditional professional development sessions, which were “sit-&-git”, meaning regular sessions were lecture-based and sedentary. Interview participants also highlighted that the trainings included new activities, which were a “breath of fresh air” and authentic.

**Table 4:**
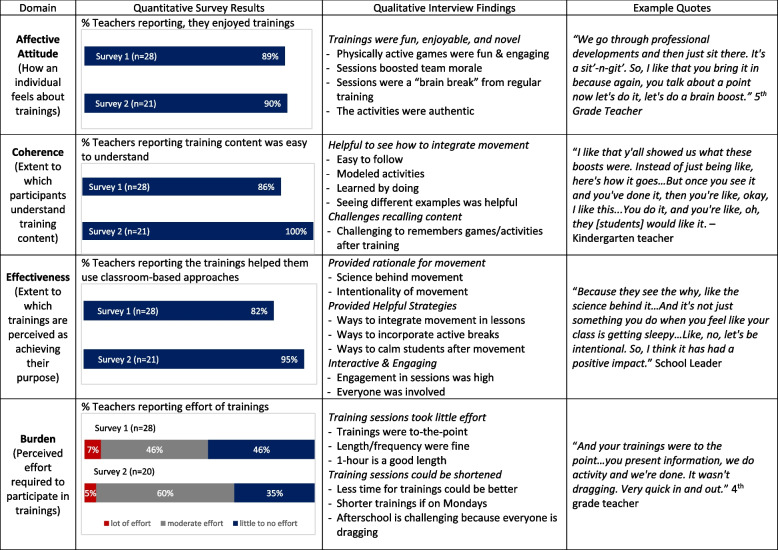
Acceptability joint display for teacher trainings

*Coherence (extent to which participants understand training content)*. Eighty-nine percent (in year 1) and 100% (in year 2) of teachers reported the training content was easy to understand (Table 4). Interview participants explained the content was easy to follow, and that they learned physically active breaks by doing them. They also highlighted how they liked that movement integration activities were modeled and that seeing different examples was helpful. Participants also highlighted that it was challenging to remember some of the physically active games (i.e., examples of physically active breaks) after the training so additional resources (e.g., handouts that have activities on them) would be helpful.

*Effectiveness (extent to which trainings are perceived as achieving their purpose)*. Eighty-five percent (in year 1) and 95.2% (in year 2) of teachers reported the trainings helped improve their use of classroom-based physical activity approaches (Table 4). Interview participants stated they learned the rationale behind movement in the classroom, including the current science and need for intentionality. Participants further stated that the trainings provided helpful teaching strategies. Specifically, they learned multiple ways to incorporate physically active breaks and integrate movement into lessons. They also felt the sessions were interactive, which helped get everyone involved and engaged throughout the sessions.

*Burden (perceived effort required to participate in trainings)*. There was mixed feedback about training burden from both surveys and interviews (Table 4). About 46% (in year 1) and 35% (in year 2) of teachers reported the trainings took little or no effort; 46% (in year 1) and 60% (in year 2) reported trainings took a moderate amount of effort, and 7.2% (in year 1) and 5% (in year 2) reported trainings took a lot of effort. Some interview participants stated that trainings were to the point, not dragging, quick, and that the 1-h session length was good. Other participants felt less time per session would be better, especially for the trainings held after the school day because it was more challenging to engage at that time.

### Newsletter acceptability

*Affective Attitude*. Of teachers who received a newsletter, 83% reported they liked the newsletters (Table 5). Interview participants stated the newsletters included helpful resources that were accessible to teachers because they had helpful links, suggestions for classroom-based approaches, and seasonal ideas. In regard to *coherence*, 83% of teachers reported the newsletters were easy to understand. Some interview participants highlighted that the newsletters were well-structured and easy to follow, and that the newsletters were simple, straight to the point, and required no extra reading. In contrast, other participants felt the newsletters were too long and that the overall format was not ideal. In regard, to effectiveness, 73.9% (year 1) and 66.7% (year 2) of teachers reported newsletters helped improve their use of classroom-based physical activity approaches. Interview participants explained that they learned about different video resources from the newsletters and that it was helpful to have something to go back to. They also felt the newsletters provided ideas that they did not need to generate on their own and that they used the resources provided in the newsletter (Table 5).

**Table 5:**
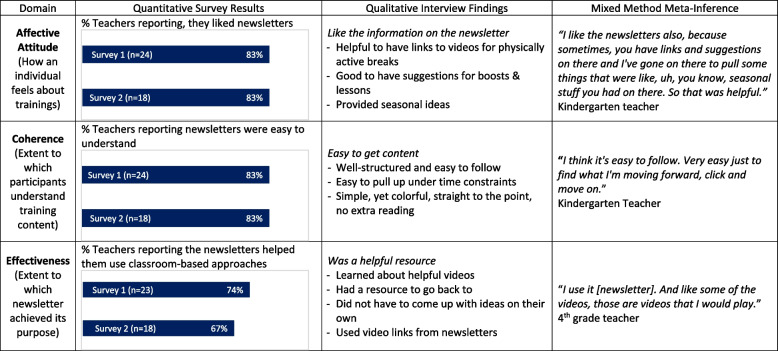
Acceptability joint display for the newsletter

The follow-up surveys did not include questions about newsletter burden because it was a passive form of distributing information

### Recommendations/additional support

All teachers (28/28) reported they would recommend MAGIC trainings and newsletters to a colleague. In year 1, 71.4% of teachers (20/28) reported MAGIC provided the right amount of support, whereas 17.9% (5/28) felt they could have used more support. In year 2, 87.5% of teachers (21/24) reported MAGIC provided the right amount of support, and 12.5% (3/24) reported “I don’t know” about needing more support. Interview participants highlighted multiple suggestions for improving MAGIC, which primarily focused on two areas: 1) improving training sessions and 2) adding strategy components.

To improve trainings, interview participants suggested the inclusion of more activities specific to each grade level and tailored activities to address specific training needs. Some primary teachers (kindergarten-2nd grade) felt the physically active breaks used in trainings were better suited for 3rd-5th grade students. Specifically, some teachers stated that they needed to adjust some physically active breaks to better align with their students’ grade and development level. Interview participants also recommended providing handouts of all physically active breaks/lessons used within the trainings to help them remember the activities. Additionally, participants suggested bringing snacks to help energize and incentivize teachers after a long school day.

Interview participants spoke of other ways to enhance MAGIC. They suggested including MAGIC resources in the school’s internal website where teachers have access to other school and district-wide resources. They also suggested creating a resource hub (e.g., google drive) that housed training materials, newsletters, and links to key websites. Participants explained that additional support could be provided by attending regularly-scheduled team meetings to further show teachers where movement opportunities could be integrated into specific sections of the curriculum. Other suggestions included providing teaching demonstrations in classrooms and recording effective teachers using classroom-based physical activity approaches.

## Discussion

This study examined the feasibility and acceptability of the MAGIC implementation strategy consisting of leadership trainings, teacher trainings, and a newsletter with educational materials. Our results indicated the leadership and teacher trainings had high attendance levels, and that a majority of teachers received and used the newsletters, supporting overall feasibility of MAGIC. Additionally, the leadership and teacher trainings in the MAGIC implementation strategy had high levels of acceptability, and the newsletters had moderate-to-high levels of acceptability. Further, all teachers reported they would recommend the trainings and newsletters to a colleague. Overall, this study provides strong evidence supporting the feasibility and acceptability of the MAGIC implementation strategy.

Professional development and staff trainings are common implementation strategies for classroom-based physical activity approaches [12, 26, 32, 33]. Existing research highlights that teacher/staff trainings can be a feasible, acceptable, and effective way to support implementation of physical activity programs in schools [21, 25, 26, 32]. However, inconsistencies across studies present challenges to understanding what makes trainings successful [36, 44]. Research suggests training that is multi-day, has comprehensive subject content, is theory informed, and includes multiple behavior change techniques has more success [36, 44]. Further, providing trainees with demonstrations of desired behaviors, opportunities to practice, feedback on performance, and goal setting appear to be promising approaches to include in training [34, 36]. Studies also highlight the need for other implementation strategies that can be used to complement trainings. Specifically, tailored ongoing supports that reinforce training content may also be necessary [22, 31, 34]. There are also higher-level implementation barriers/facilitators (e.g., leadership support, culture) that often need to be addressed, which extend beyond what teacher training supports [21, 45–47].

The leadership and teacher trainings in MAGIC included many of the recommended characteristics. We developed MAGIC using Implementation Mapping, which guided the use[26] of Social Cognitive Theory and the R = MC^2^ heuristic [39, 40]. Implementation Mapping also helped identify change methods (i.e., theoretical techniques, similar to behavior change techniques) to address implementation determinants. As a result, MAGIC used multiple change methods throughout the training, including modeling, experiential learning, guided practice, and goal setting (among others) [27, 48]. Participants highlighted how they liked “learning by doing” and seeing demonstrations, which contributed to MAGIC’s high ratings of acceptability.

Another key feature of Implementation Mapping is the partner driven approach to co-develop the implementation strategy and materials [26]. Using a co-development process is critical for ensuring implementation strategies meet partner needs and work within the school context [34, 49]. Our school and district partners helped guide decisions about the scheduling and duration of teacher trainings, which resulted in three sessions (about 1-h each). We also found that providing flexible scheduling and integrating trainings into existing meetings helped lead to high attendance levels and were likely important contributors to the feasibility of MAGIC.

MAGIC also included leadership training, which was designed to improve leadership support throughout the implementation phases (adoption, implementation, and maintenance). Despite the importance of leadership support, leadership trainings are not as commonly reported/used compared to teacher training. We found that working with leaders first to get buy-in, was important prior to conducting teacher trainings. This ensured that leaders understood the rationale for incorporating physical activity throughout the school day and positioned leaders to support teachers at the onset. In total, there were four leadership trainings that were about 45–60 min in duration. Our results indicated this was a feasible training dose for leaders. Similar to the teacher training, conducting the leadership sessions during regularly scheduled meetings, and providing flexible scheduling options aided attendance and completion. Additionally, incorporating physical activity into the leadership sessions (i.e., using experiential learning and modeling approaches) contributed to the acceptability.

Past research indicates that providing tailored, ongoing support that reinforces training content is often necessary [22]. This ongoing support was provided through monthly newsletters in the MAGIC implementation strategy. Newsletters have been used in previously, and offer an avenue to reach teachers and staff with key information and resources for ongoing implementation [31]. Our results indicate the MAGIC newsletters were a feasible and acceptable strategy component. Further, participants highlighted key features such as the colorful, simple design, and links to helpful online resources.

Overall, our findings highlight key points to consider when using trainings and newsletters. First, integrating training into existing meetings can help attendance. This integrated approach requires flexible scheduling, but can reduce the burden of additional afterschool meetings. Second, incorporating physical activity into training sessions is a critical element. Consistent with previous studies [22, 34], teachers and leaders consistently stated that learning with physical activity helped build buy-in and motivation. This was because teachers and leaders could experience firsthand how movement-based approaches helped learning, and thus could see how the approaches would benefit their students. Third, to reach teachers with additional educational content, multiple distribution approaches are likely necessary. Emailed newsletters can be a baseline approach, but providing additional paper-based handouts, prompts about resources, and other ways to access resources (e.g., a google drive) can help ensure that teachers receive information. Fourth, trainings should not be limited to classroom teachers. Providing leadership training is critical given leaders help drive implementation efforts and maintain support for teachers [47].

Even though there was strong evidence of feasibility and acceptability, there were suggested improvements to the MAGIC implementation strategy. Training sessions were held with all school staff simultaneously, which made it difficult to tailor content for each grade. For example, when demonstrating physically active learning approaches, providing examples for each grade level can be a helpful way to address all teachers within a large group. Multiple demonstrations within the same training session could be challenging given time constraints. Thus, explaining how activities could be adapted for each grade, or providing additional handouts highlighting adaptations may be alternative avenues. Most teachers felt the trainings and newsletters provided the right amount of support. However, for teachers and staff that need (or want) additional support, this could be provided through grade-level team meetings (i.e., professional learning communities) [46] or providing access to more comprehensive resources through a website or information portal (e.g., google drive). Overall, there can be high levels of variability between schools [50], so multiple avenues to access content, and providing materials with different levels of implementation support can help meet school needs.

### Limitations and strengths

There are study limitations to consider. First, the feasibility and acceptability testing were conducted with one elementary school. Given the variation among schools, additional testing is necessary. This study also used self-report surveys to examine the different aspects of acceptability. The use of self-report surveys may lead to recall and/or social desirability bias. Further, the surveys were limited to classroom teachers. While classroom teachers were the intended population for the teacher training, all staff (including leaders) within the school participated in trainings. Thus, obtaining survey feedback from all school staff (e.g., school leaders, library, art, music, physical education teachers) could have been helpful to better understand perspectives beyond the teachers of focus. Additionally, obtaining survey data from school leaders could have enhanced our understanding of acceptability of leadership training sessions. Lastly, we did not collect other background variables such as teacher’s attitudes and participation in physical activity, which could provide valuable context to findings.

Despite the limitations there were numerous strengths. The teacher surveys had high response rates (100% in year 1 and 96% in year 2) and there were high levels of participation in the teacher and leadership interviews. Additionally, the mixed methods design included multiple points of integration. Notably, the survey and interview samples were overlapping, the survey and interview guide questions were complementary, qualitative and quantitative data were merged during analyses. We also presented results using joint displays to facilitate comparisons and provide an in-depth understanding of feasibility and acceptability. The theoretical framework for acceptability helped guide survey questions and domains for analysis. Lastly, our results align with previous studies that highlight effective training characteristics, and add to the literature by providing valuable acceptability and feasibility information for training school leaders [34, 36, 44].

## Conclusion

Implementation strategies for evidence-based physical activity approaches in schools are critical for supporting children’s health, academics, behavior, and overall well-being. Our study provides evidence supporting the feasibility and acceptability of MAGIC, an implementation strategy for classroom-based physical activity approaches in elementary schools. Our findings highlight the importance of integrating implementation support into existing school activities (e.g., existing meetings and distribution approaches), educating teachers/staff through the demonstrated use of physically active breaks and lessons, and working first with school leaders to help support teachers. Future work should examine MAGIC among a larger sample of schools, including comparisons across grade levels, and its impact on implementation outcomes and student physical activity levels both in the short- and long-term. Supporting the implementation of school-based physical activity approaches has great potential to ensure students, schools, and staff receive the many benefits.

## Supplementary Information

Supplementary Material 1.

Supplementary Material 2.

Supplementary Material 3.

## Data Availability

The datasets used and/or analyzed during the current study are available from the corresponding author(s) on reasonable request.

## Abbreviation

MAGIC: Movement for Academic Growth In Classrooms
