# Supplementary material for: Feasibility and acceptability of an implementation strategy to enhance use of classroom-based physical activity approaches in elementary schools: a mixed methods study
Source: BMC Public Health. 2025 Nov 27;26:36. doi: 10.1186/s12889-025-25333-0 (PMC12763913; doi:10.1186/s12889-025-25333-0)
Supplement: Supplementary file 1 — Supplementary Material 1. [file 12889_2025_25333_MOESM1_ESM.docx]

Table 1: Implementation Strategy Component Specification

|  | **Leader Trainings** | **Teacher Trainings** | **Newsletters** |
| --- | --- | --- | --- |
| **Actors**  (Who enacts the strategy) | Study Team & District Wellness Coordinator | Study Team, District Wellness Coordinator, & School Leaders/Staff | Study Team &  District Wellness Coordinator and School Leaders |
| **Actions**  (Actions that need to be enacted) | S1: Setting goals and communication plan to teachers  S2: Creating a culture to support movement and learning by engaging champions, providing reinforcement, modeling, and using morning movement opportunities  S3: Reviewing and reflecting on use of culture building approaches throughout the year  S4: Creating a sustainability plan | S1: Using physically active breaks and addressing common barriers  S2: Using physically active lessons as a general practice and ways to integrate movement into existing curricula  S3: Maintaining physically active breaks and lessons, and helping new teachers | Distribute newsletter  (Newsletter reinforces training content and provides links to resources) |
| **Action Target**  (Targets of implementation) | School Leadership Team | School Staff (with a focus on classroom teachers) | School staff (with a focus on classroom teachers) |
| **Temporality**  (When strategy is used) | S1: beginning of school year 1  S2: fall school year 1  S3: winter school year 1  S4: beginning of school year 2 | S1: fall school year 1  S2: winter school year 1  S3: fall of school year 2 | Monthly distribution after first teacher training begins |
| **Dose**  (Dosage of implementation) | 4 in-person sessions (45-60 mins) | 3 in-person sessions (60 mins) | 1 newsletter/month |
| **Implementation outcomes**  (Outcome likely to be impacted) | Acceptability of classroom-based approaches  Implementation Fidelity | Acceptability of classroom-based approaches  Implementation Fidelity | Acceptability of classroom-based approaches  Implementation Fidelity |
| **Justification**  (Justification for choice of strategy) | Improve knowledge, attitudes, skills/self-efficacy, overcoming barriers, leadership support, culture, priority | Improve knowledge, attitudes, skills/self-efficacy, overcoming barriers, intraorganizational relationships, culture, priority | Improve knowledge, attitudes, skills/self-efficacy, overcoming barriers, leadership support, intraorganizational relationships, culture, priority |

S: Session
