# Supplementary material for: Feasibility and acceptability of an implementation strategy to enhance use of classroom-based physical activity approaches in elementary schools: a mixed methods study
Source: BMC Public Health. 2025 Nov 27;26:36. doi: 10.1186/s12889-025-25333-0 (PMC12763913; doi:10.1186/s12889-025-25333-0)
Supplement: Supplementary file 2 — Supplementary Material 2. [file 12889_2025_25333_MOESM2_ESM.docx]

Supplemental Table 2 – Survey and Qualitative Interview Questions

| Component | Domain | Survey Questions | Interview Questions |
| --- | --- | --- | --- |
| Teacher Training | Affective Attitude | I enjoyed attending the MAGIC training sessions* | How did you feel about the training sessions?  Probes:   - What worked well? - What did not work well? - What recommendations? |
|  | Coherence | Content of the training was easy to understand* |  |
|  | Effectiveness | The MAGIC training sessions helped improve my use of brain boosts or physically active lessons* |  |
|  | Burden | How much effort did it take to participate in the MAGIC training sessions?** |  |
| Newsletter | Affective Attitude | I liked the MAGIC newsletters* | How did you feel about the newsletters?  Probes:   - What worked well? - What did not work well? - What recommendations? |
|  | Coherence | The content in the MAGIC newsletters was easy to understand* |  |
|  | Effectiveness | The MAGIC newsletters helped improve my use of brain boosts or physically active lessons* |  |
| Leadership Training |  | No leadership survey | How did you feel about the leadership training sessions?  Probes:   - What worked well? - What did not work well? - What recommendations? |

*, used a 5-point Likert Scale (strongly disagree, somewhat disagree, neither agree nor disagree, somewhat agree, strongly agree)

**, used a 5-point Likert-type scale (no effort at all, a little effort, a moderate effort, a lot of effort, huge effort)
