# Supplementary material for: Feasibility and acceptability of an implementation strategy to enhance use of classroom-based physical activity approaches in elementary schools: a mixed methods study
Source: BMC Public Health. 2025 Nov 27;26:36. doi: 10.1186/s12889-025-25333-0 (PMC12763913; doi:10.1186/s12889-025-25333-0)
Supplement: Supplementary file 3 — Supplementary Material 3. [file 12889_2025_25333_MOESM3_ESM.docx]

Supplemental Table 3. Complete Distributions for Acceptability of Training and Newsletter

|  | | Mean (Standard Deviation) | |
| --- | --- | --- | --- |
|  |  | Survey 1 | Survey 2 |
| Teacher Training | Affective Attitude* | 4.5 (0.7) | 4.7 (0.7) |
|  | Coherence* | 4.7 (0.7) | 4.7 (0.5) |
|  | Effectiveness* | 4.5 (0.7) | 4.6 (0.6) |
|  | Burden** | 2.4 (0.9) | 2.6 (0.8) |
| Newsletter | Affective Attitude* | 4.4 (0.8) | 4.4 (0.8) |
|  | Coherence* | 4.5 (0.8) | 4.6 (0.8) |
|  | Effectiveness* | 4.3 (0.9) | 4.2 (0.9) |

*, used a 5-point Likert Scale (strongly disagree, somewhat disagree, neither agree nor disagree, somewhat agree, strongly agree)

**, used a 5-point Likert-type scale (no effort at all, a little effort, a moderate effort, a lot of effort, huge effort)
